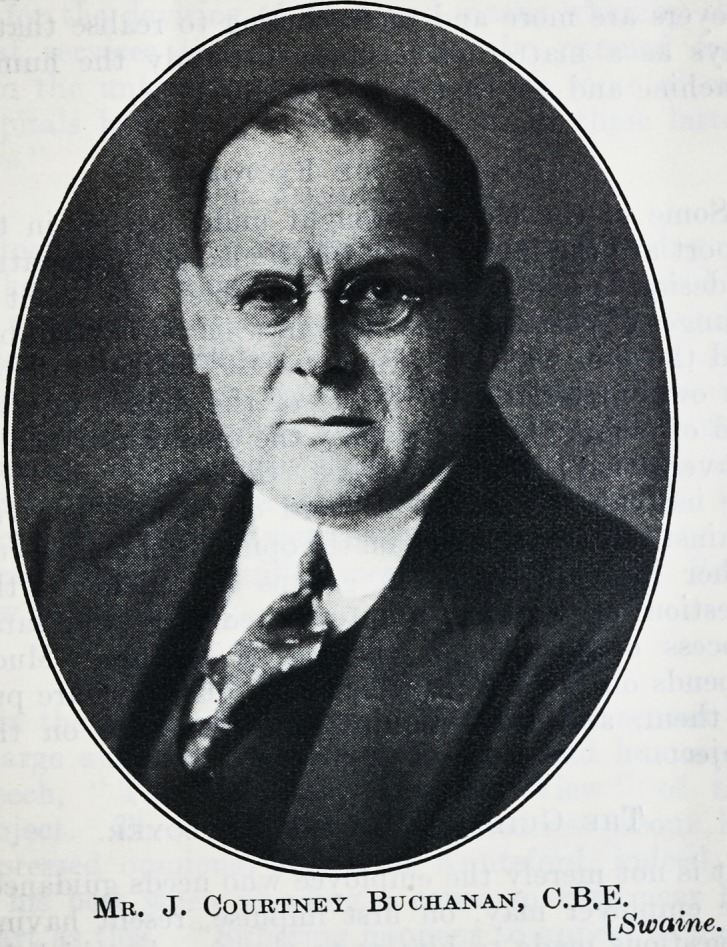# Hospital Men of Mark: Mr. J. Courtney Buchanan, C.B.E.

**Published:** 1924-06

**Authors:** 


### HOSPITAL MEN OF MARK.

### MR. J. COURTNEY BUCHANAN, C.B.E.

Born in 1877, the grandson of the Vicar of Herne, near Canterbury, Mr. Buchanan, who lost both parents when he was only seven years old, was educated at Christ's Hospital, where he became a Grecian, as members of the top form are called. Few schools have been more identified with men of letters, and it was therefore fitting that Mr. Buchanan should, after matriculation, have won a scholarship at London University for Classics and English Literature. Under this scholarship Mr. Buchanan was due to read Political Science and Economics. After

Mr. J. Courtney Buchanan, C.B.E.

[*Swaine.*

two years he decided to resign the Scholarship and read for a professional qualification. He therefore entered at Lincoln's Inn with a view to being called to the Bar. He combined his legal studies with work in the secretarial department of St. Bartholomew's Hospital, where he remained until 1906, when he received his first important hospital appointment as secretary to the Building Committee of the Bolingbroke Hospital. The additions, which had been begun in 1901, were continued in 1906 and lasted for three years, during which the hospital obtained a charter of incorporation. It was an onerous time for all concerned, and, no doubt, a very valuable experience for Mr. Buchanan. Two years later the secretaryship of the Metropolitan Hospital fell vacant, and Mr. Buchanan received this post, which, if the size of the two institutions be compared, about doubled his responsibilities. Here, again, reconstruction was in progress.

Wider questions, following the issue of the Majority and Minority Reports of the Poor Law Commission, and the prospect of Health Insurance by the State, were then beginning to attract attention, and in 1910 Mr. Buchanan read a paper before the Incorporated Association of Hospital Officers on "The Functions of the Voluntary Hospitals in relation to the Proposed Public Assistance Authority." This was issued as a pamphlet in the following year, and in it he argued that hospital finances would, sooner or later, have to be supplemented by State or municipal subventions if they were to continue the same work; discussed various plans for co-operative action; pointed out the growing tendency for the work of the hospital to be consultative; and dealt with the needs of paying patients and the desirability of encouraging patients to contribute to the cost of their treatment. In this pamphlet, too, he emphasized the need for hospitals to define their position—in short, to have a policy. "Unless," he added, "the voluntary hospitals define their position, their position will be defined for them from outside." Subsequent events have clearly demonstrated the foresight of this remark. An even more curious example of "intelligent anticipation" is seen in the suggestion that it might not be impossible or, indeed, very difficult to devise "a kind of hospital or charitable poll-tax, on a graduated scale, for spectators at theatres and places of amusement." Since then we have had the Entertainments Tax! In 1911 Mr. Buchanan read a paper on "Voluntary Hospitals and the Insurance Bill" before the British Medical Association Conference at the famous Insurance Bill meeting of 1911. Later in that year Mr. Buchanan read at the Conference at Manchester the first of the papers he has read before the British Hospitals Association.

The interests indicated by these activities naturally led to active support (as a member of the Council from 1911) of the British Hospitals Association, an essential, if, in the past, somewhat unwieldly body, whose members and institutions were widely separated, with different problems, different conditions and different sources of income. It was not till 1915 that Mr. Buchanan became honorary secretary to the Association, and it has been during his term of office that it has been divided administratively into Regional Committees and has gained so enormously in usefulness and prestige. In 1920 Mr. Buchanan was created a C.B.E. for his hospital work during the war, and in the same year was elected secretary to the Cancer Hospital. It would seem that Mr. Buchanan is always to be associated with constructive work, for again at the Cancer Hospital important additions and improvements are in hand. In October last Mr. Buchanan was elected president of the Hospital Officers' Club for the next three years.

Before his marriage Mr. Buchanan used to play Rugby football in the winter and, in his spare time, to row all the year round. Latterly, when there has been a holiday, he has found recreation in golf; but he still cherishes a "pot" that he won for swimming at Broadstairs Regatta in 1893. He enjoyed a successful year of office as Master of the Nosocomia (which, being interpreted, means "Hospitals") Lodge during 1922-3; and since its foundation has served as Treasurer of the Nosocomia Lodge of Instruction.

## Figures and Tables

**Figure f1:**